# SNP-based molecular diagnostic platform: rapid single-step identification of *Theileria annulata* and its buparvaquone-resistant strains

**DOI:** 10.1186/s13071-025-06884-y

**Published:** 2025-07-01

**Authors:** Jin Che, Yijun Chai, Shuaiyang Zhao, Jinming Wang, Jianxun Luo, Guiquan Guan, Hong Yin, Wei Li

**Affiliations:** 1 College of Veterinary Medicine, Northeast Agricultural University, Harbin, 150006 China; 2 State Key Laboratory for Animal Disease Control and Prevention, Key Laboratory of Veterinary Parasitology of Gansu Province, Lanzhou Veterinary Research Institute, Chinese Academy of Agricultural Sciences, Lanzhou, 730046 China; 3 Jiangsu Co-Innovation Center for the Prevention and Control of Important Animal Infectious Disease and Zoonosis, Yangzhou University, Yangzhou, 225009 China

**Keywords:** Buparvaquone resistance, Cytochrome b, Real-time PCR, SNP genotyping, *Theileria annulata*

## Abstract

**Background:**

*Theileria annulata*, a tick-borne protozoan that causes tropical theileriosis, poses a serious threat to livestock production in endemic regions. The emergence of resistance to buparvaquone, the primary chemotherapeutic treatment, has been attributed to acquired mutations in the cytochrome b (*Cytb*) gene, with identical resistance-associated polymorphisms observed in both laboratory-adapted strains and field isolates from China.

**Methods:**

A dual probe-specific real-time polymerase chain reaction (PCR) assay was developed to detect point mutations in the *Cytb* gene. The specificity, sensitivity, and reproducibility of the assay were validated, and its field applicability was evaluated via cattle blood samples (*n* = 531) collected from five endemic Chinese provinces.

**Results:**

Six point mutations were identified in the *Cytb* gene, and the developed dual probe-specific real-time PCR assay simultaneously detected *T. annulata* infection and distinguished between the buparvaquone-sensitive and buparvaquone-resistant genotypes. The assay demonstrated a detection limit of 1 × 10^1^ copies/μl, high specificity, and satisfactory repeatability, with results consistent with those of Sanger sequencing. Field screening revealed a 21.7% (115/531) prevalence of *T. annulata* and a 4.3% (23/531) occurrence of resistant genotypes. Moreover, two-dimensional scatterplot visualization enabled clear genotype discrimination without post-PCR processing.

**Conclusions:**

The developed dual probe-specific real-time PCR assay enables efficient detection of buparvaquone-resistant genotypes, providing important implications for guiding the treatment of tropical theileriosis and enhancing epidemiological surveillance of emerging resistance in endemic regions.

**Graphic abstract:**

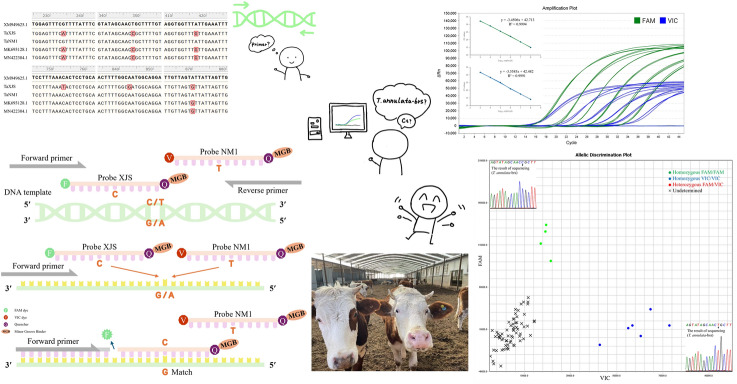

**Supplementary Information:**

The online version contains supplementary material available at 10.1186/s13071-025-06884-y.

## Background

*Theileria annulata* is a highly pathogenic tick-borne protozoan parasite and the primary causative agent of bovine tropical theileriosis. This widespread disease poses serious threats to cattle health and results in substantial economic losses across Southern Europe, North Africa, and Asia, where approximately 250 million cattle are at risk [1]. The presence of multiple cattle breeds and buffaloes remains a major impediment to livestock development in many African and Asian regions [2, 3]; its complex epidemiology involves intricate interactions among hosts, pathogens, and vectors, which are further influenced by livestock management practices, agroecological factors, and sociopolitical conditions [4]. Buparvaquone is widely used to treat bovine tropical theileriosis owing to its high efficacy during the early stages of infection [5].

Currently, resistance to buparvaquone in *T. annulata* is attributed primarily to mutations in the *TaPIN1* gene [6], which has also been widely used as a molecular target for detecting drug resistance. Several studies have reported treatment failure in *T. annulata* infections associated with these mutations, including a recent identification of a similar *TaPIN1* mutation in a Chinese isolate [7]. A TaqMan probe-based real-time PCR assay was previously developed to investigate the prevalence of *TaPIN1* mutations in Chinese isolates, providing a specific, sensitive, and reproducible method for detecting resistant *TaPIN1* variants [8]. In addition to *TaPIN1*, mutations in the cytochrome b (*Cytb*) gene have emerged as key markers for buparvaquone resistance in *T. annulata* [9]. These resistance mechanisms are primarily linked to nonsynonymous *Cytb* mutations [10], such as methionine-to-isoleucine substitutions at position 128 [11], which disrupt drug binding within the mitochondrial electron transport chain and reduce treatment efficacy. Mutations at critical binding sites, particularly the Q_01_ and Q_02_ regions of *Cytb*, substantially diminish buparvaquone activity, thereby conferring resistance in infected cattle [7, 12].

To address this diagnostic challenge, advanced molecular techniques such as the TaqMan SNP real-time PCR assay utilizing minor groove binder (MGB) probes have been developed [13], offering increased sensitivity and specificity [14, 15]. TaqMan SNP probes have been successfully applied in various fields, including the identification of fluoroquinolone-resistant *Mycoplasma bovis* and oseltamivir-resistant influenza A, underscoring their versatility and precision in the detection of drug-resistant pathogens [16, 17].

In this study, we aimed to construct a dual probe-specific real-time PCR assay employing TaqMan MGB probes to target single-nucleotide polymorphisms (SNPs) in the *Cytb* gene for the simultaneous detection of *T. annulata* infection and buparvaquone resistance. By leveraging the high specificity of MGB probes for key drug resistance mutations, this approach integrates pathogen detection and resistance profiling into a single, streamlined assay. This method not only improves diagnostic accuracy and efficiency but also provides a robust tool for managing bovine tropical theileriosis, offering substantial improvements in field diagnostics and enabling timely, evidence-based disease control strategies in endemic regions.

## Methods

### Cells and blood samples

TaNM1 and TaXJS, two bovine lymphocyte cell lines infected with *T. annulata*, were used in this study. Specifically, TaNM1 harbors a buparvaquone-susceptible strain (*T. annulata*-bss), whereas TaXJS harbors a buparvaquone-resistant strain (*T. annulata*-brs) [18]. The genomic DNA of *T. sinensis*, *T. orientalis*, *B. bigemina*, and *B. bovis* were provided by the Vector and Vector-borne Diseases (VVBD) Team, Lanzhou Veterinary Research Institute, Chinese Academy of Agricultural Sciences (CAAS), China, to validate the specificity of the assay.

Field cattle blood samples (*n* = 531) were collected from five different provinces in China, and the DNA of the blood samples was extracted via the TIANamp blood DNA kit (Tiangen Biotech Co. Ltd; Beijing; China) according to the manufacturer’s protocol.

### TaqMan-MGB probes and primers

The primers TaCytb-F/R used to amplify the full-length sequences of the *Cytb* gene (1092 bp) of TaNM1 and TaXJS were designed according to the standard protocol [19]. The published primer, Cytb-F/R, was used for the detection of *T. annulata* infection in the PCR assay (Additional File 1) [20]. The Ankara strain (XM949625.1) was used as reference sequence of the *T. annulata Cytb* gene for alignment with the sequenced TaNM1, TaXJS, and reported mutated strains [12, 21]. The 115-bp fragment of the *T. annulata Cytb* gene was amplified via primers (SNP-F/R) and TaqMan-MGB probes (Probe-NM1/XJS). Both the primers and probes were designed by SnapGene according to the standard protocol [22]. The probes were labeled with VIC/FAM dyes recognizing the ACT/ACC genotype, respectively. The details are presented as Table 1. The primers and probes were custom synthesized through outsourcing from Beijing Tsingke Biotech Co., Ltd.

**Table 1:** Probes and primers used in the present study

Primer/probe	Sequence (5′–3′)	Product size (bp)
TaCytb-F	ATGAATTTGTTTAACTCACATTTGC	1092 bp
TaCytb-R	TTATGCACGAACTCTTGCAGAGTC	
Cytb-F	TGGTCTTGGTATTCTGGTGTT	393 bp, Junlong et al. [20]
Cytb-R	GCCAATGGATTTGAACTTCC	
SNP-F	TGGTCTTGGTATTCTGGTGTT	115 bp
SNP-R	AACCACCTATGACTGTAGCT	
Probe-NM1	VIC-AGTATAGCAACTGCTT-MGB	
Probe-XJS	FAM-AGTATAGCAACCGCTT-MGB	

### Construction of standard plasmids

The standard plasmid was constructed from the complete *Cytb* genes of *T. annulata*-bss and *T. annulata*-brs. The *Cytb* genes were amplified, cloned into the pGEM-T Easy vector (Progema, USA), and sequenced by Beijing Tsingke Biotech Co., Ltd. (Beijing, China), with the sequences subsequently validated [23]. The standard plasmids were labeled T-XJS and T-NM1, respectively (Additional File 2).

The T-XJS and T-NM1 plasmids were continuously diluted tenfold from 1 × 10^8^ to 1 copies/μl. The copy number was calculated via the formula [copy number = recombinant plasmid concentration × Avogadro constant × 10^–9^/(660 bp × total size of the pGEM-T Easy vector)] [23].

### Reaction conditions and optimization

The dual probe-specific real-time PCR assay was developed, and the final reaction mixtures consisted of 10 μl of 2 × ChemQ Geno-SNP Probe Master Mix (Vazyme, China), 1.8 μl of each primer (10 μM), 2 μl of DNA template, and 0.4 μl of each probe. For screening optimization, probe-NM1 and probe-XJS were systematically evaluated via stepwise concentration gradients (50–400 nM). Incremental titration at 50 nM intervals was applied to establish dose‒response relationships. Finally, nuclease-free water was added to a final volume of 20 μl. Amplification was performed on a QuantStudio 5 instrument (Applied Biosystems, USA), and the two-step protocol conditions were as follows: 30 s for DNA denaturation at 95 °C followed by 45 cycles of 95 °C for 10 s, 60 °C for 30 s. The fluorescence signal was collected at the end of each cycle.

### Establishment of standard curves and repeatability of tests

Different concentrations of plasmid ranging from 1 × 10^8^ to 1 × 10^3^ copies/μl were used as templates for dual probe-specific real-time PCR to generate standard curves, on the basis of the calculated *R*^2^ (correlation coefficient), *E* value (amplification efficiency), and standard equation. Three independent tests were performed, and each reaction was repeated three times. The coefficient of variation (CV) of the Ct values was used to estimate repeatability.

### Sensitivity and specificity tests

The sensitivity of the standardized method in this study was assessed by dual probe-specific real-time PCR amplification with plasmid templates ranging from 1 × 10^3^ to 1 copy/μl. To assess the specificity of the test, the genomic DNA of *T. sinensis*, *T. orientalis*, *B. bigemina*, and *B. bovis* was used as a template.

### Dual probe-specific real-time PCR assay for the detection of *T. annulata* in field blood samples

We collected 531 field cattle blood samples from five different provinces of China and applied our assay to detect *T. annulata*-bss/brs. Positive samples were sequenced and validated against sequences from the NCBI database. The comparison tests were performed via a previously published method to detect *T. annulata*-brs [8] and a method in which the *Cytb* gene was targeted to detect *T. annulata* [20].

## Results

### Detection of SNPs

**Fig. 1:**
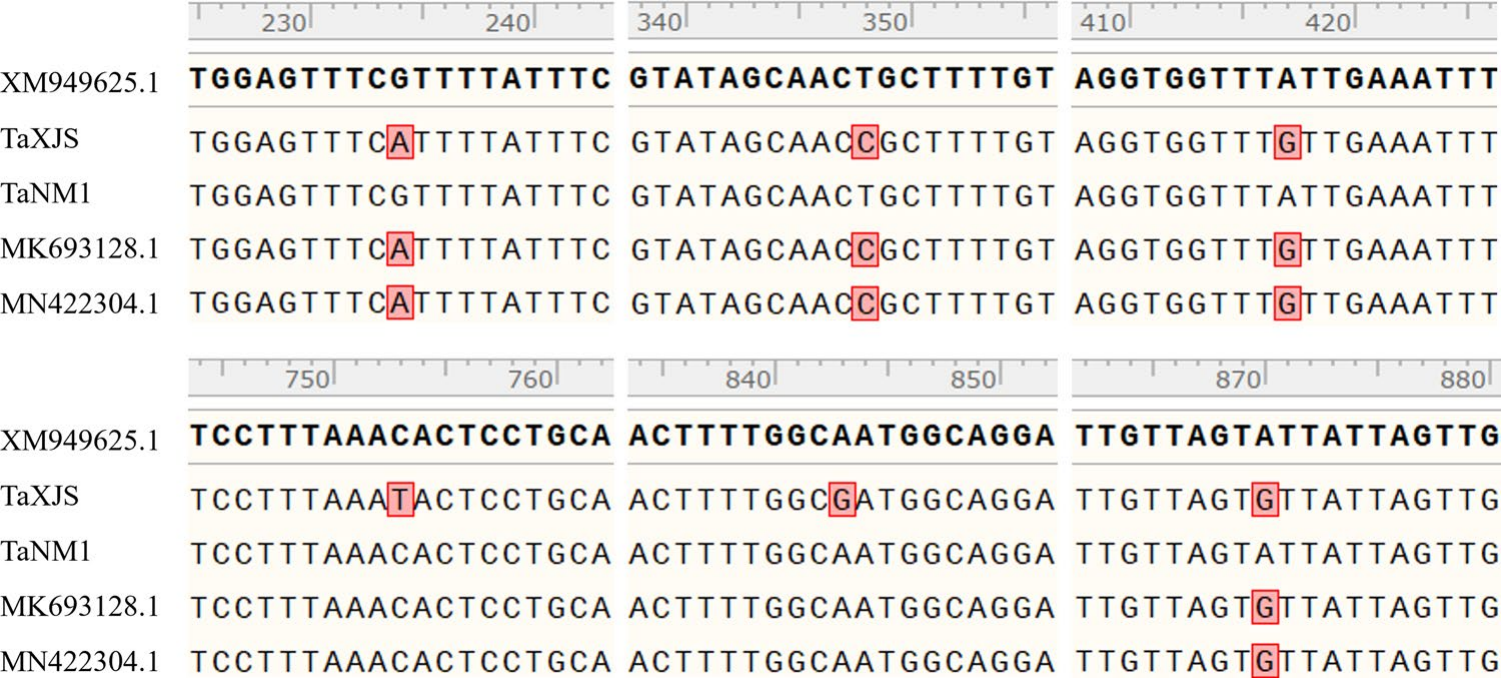
Nucleotide alignment of the *T. annulata Cytb* gene. The mutated nucleotides are marked in red

Six point mutations located at positions 234, 348, 417, 753, 843, and 870 of the *Cytb* gene were identified (Fig. 1). The point mutations located at positions 234, 348, 417, and 870 have been demonstrated in previous studies [12, 21, 24], whereas positions 753 and 843 were newly identified in this study.

### Primer probe specificity validation and concentration screening

**Fig. 2:**
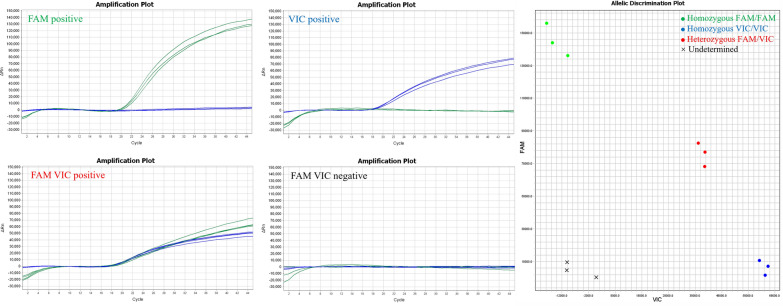
Primer probe specificity validation. The green dots represent the homozygous FAM/FAM, and the template was T-XJS. The blue dots represent the homozygous VIC/VIC, and the template was T-NM1. The red dots represent the heterozygous FAM/VIC, and the template was a mixture of T-XJS and T-NM1. The black X represents undetermined, and the template was nuclease-free water

**Fig. 3:**
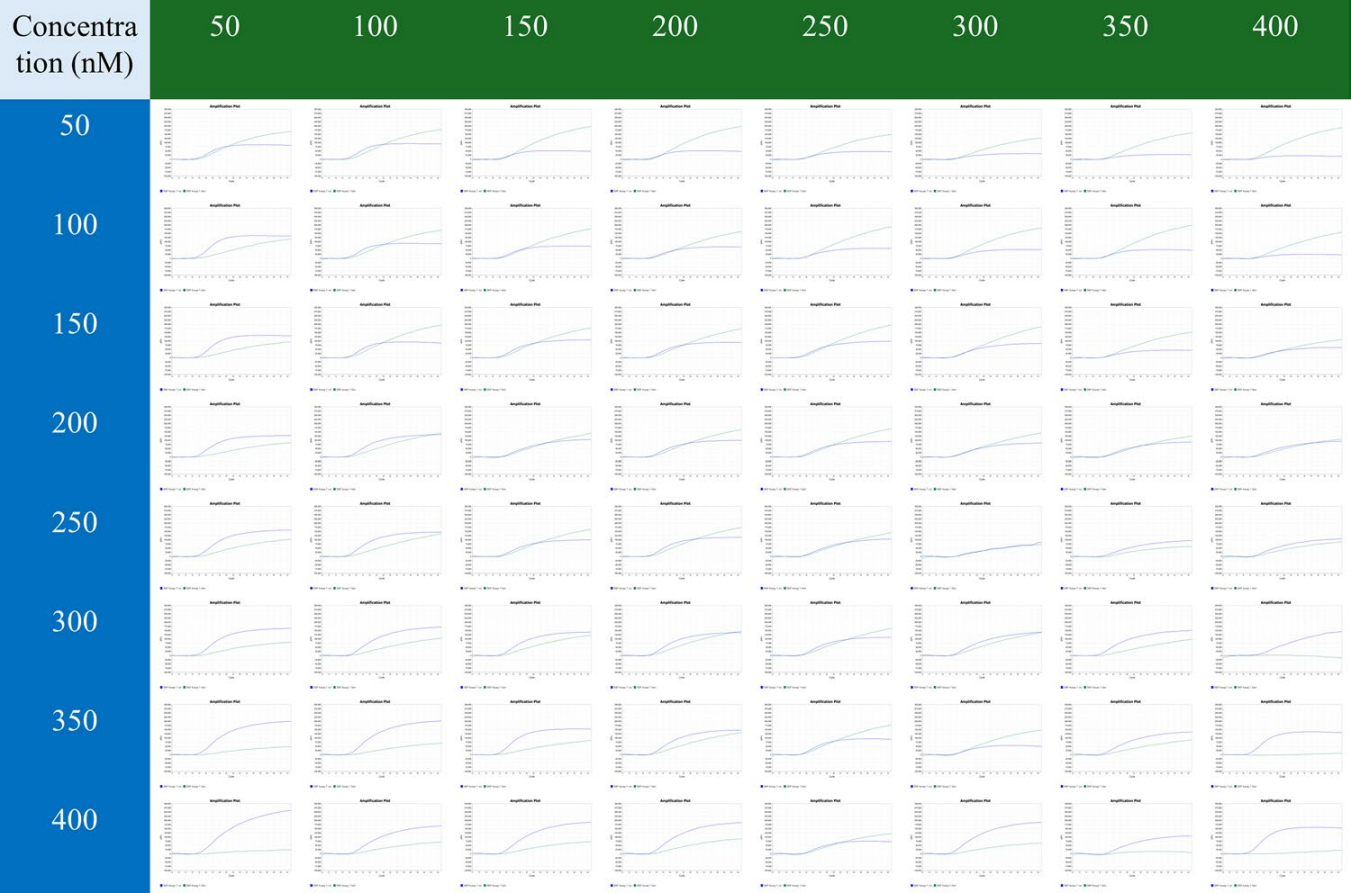
Probe concentration screening. The numbers in the green and blue parts represent the concentrations of probe-XJS and probe-NM1, respectively

A dual probe-specific real-time PCR genotyping assay was performed to validate the specificity of the primers and probes. Clear genotyping results were obtained, and no nonspecific amplification was observed (Fig. 2). On the basis of the Ct value (18.59) and analysis of the amplification curves, the optimal concentrations for probe-XJS and probe-NM1 were 150 nM and 200 nM, respectively (Fig. 3).

### Establishment of standard curves and repeatability tests

**Fig. 4:**
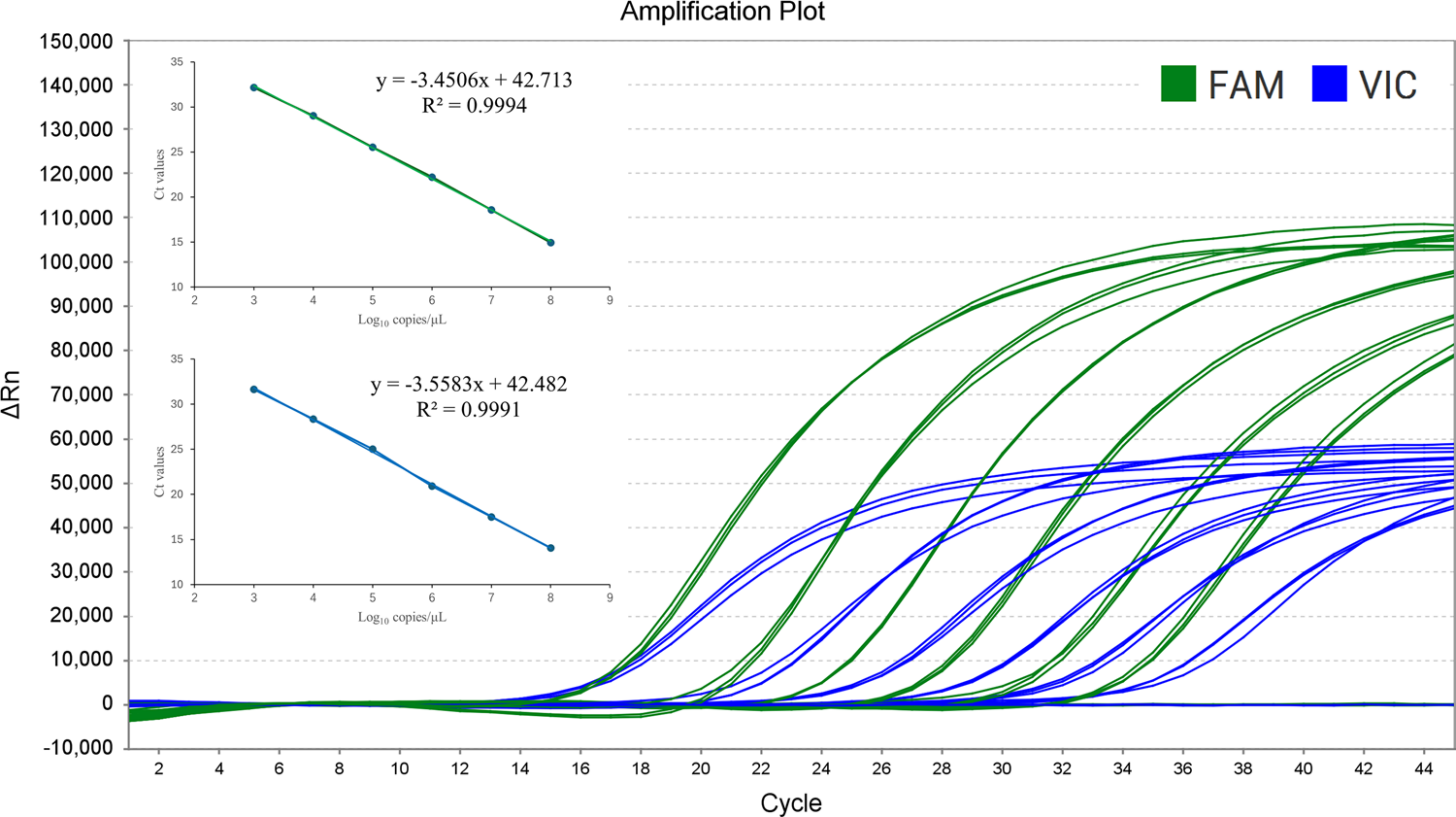
Standard curve established. Dual probe-specific real-time PCR amplification with serial dilutions of T-XJS (green color, FAM fluorescence channel) and T-NM1 (blue color, VIC fluorescence channel)

The standard curves for T-XJS (*y* = − 3.4506*x* + 42.713, *R*^2^ = 0.9994, *E* value = 94.90%) and T-NM1 (*y* = − 3.5583*x* + 42.482, R^2^ = 0.9991, *E* value = 91.00%) demonstrated strong linear correlations between the Ct values and corresponding copy numbers (*R*^2^ > 0.999) of both amplifications (Fig. 4).

### Repeatability of the real-time PCR assay

Repeatability was evaluated by testing different concentrations of T-XJS and T-NM1. The intra-assay and inter-assay CVs of the Ct values ranged from 0.10% to 0.92% and from 0.23% to 1.93%, respectively (Table 2). These results indicate that the method established in this study has satisfactory repeatability.

**Table 2:** Repeatability test of the real-time PCR assay

Pathogen	Concentration (copies/*μ*L)	Intra-assay CV		Inter-assay CV	
		*X* ± SD	CV (%)	*X* ± SD	CV (%)
TaXJS	10^8^	14.93 ± 0.03	0.21	14.87 ± 0.12	0.80
	10^7^	18.55 ± 0.03	0.18	18.51 ± 0.08	0.44
	10^6^	22.21 ± 0.03	0.15	22.19 ± 0.05	0.23
	10^5^	25.52 ± 0.10	0.38	25.49 ± 0.10	0.41
	10^4^	29.01 ± 0.10	0.35	28.93 ± 0.17	0.59
	10^3^	32.15 ± 0.09	0.29	32.09 ± 0.14	0.43
TaNM1	10^8^	14.05 ± 0.06	0.40	14.29 ± 0.25	1.78
	10^7^	17.50 ± 0.16	0.92	17.59 ± 0.20	1.16
	10^6^	20.91 ± 0.09	0.42	21.15 ± 0.41	1.93
	10^5^	25.00 ± 0.02	0.10	24.94 ± 0.11	0.46
	10^4^	28.34 ± 0.05	0.19	28.43 ± 0.16	0.57
	10^3^	31.64 ± 0.20	0.64	31.51 ± 0.28	0.90

### Sensitivity of the real-time PCR assay

**Fig. 5:**
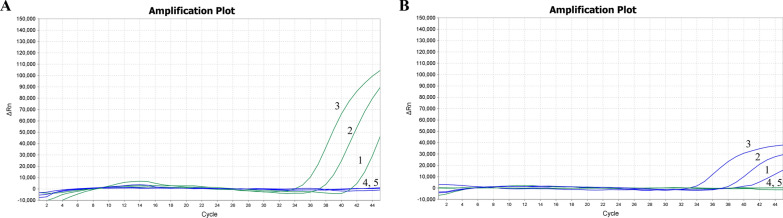
Sensitivity test of the dual probe-specific real-time PCR assay. **A** *T. annulata*-brs, **B** *T. annulata*-bss, 1: 1 × 10^1^ copies/μl, 2: 1 × 10^2^ copies/μl, 3: 1 × 10^3^ copies/μl, 4: 1 copies/μl, 5: negative control

The results indicated that the method established in this study could detect *T. annulata*-bss and *T. annulata*-brs at concentrations as low as 1 × 10^1^ copies/μl (Fig. 5).

### Specificity of the real-time PCR assay

**Fig. 6:**
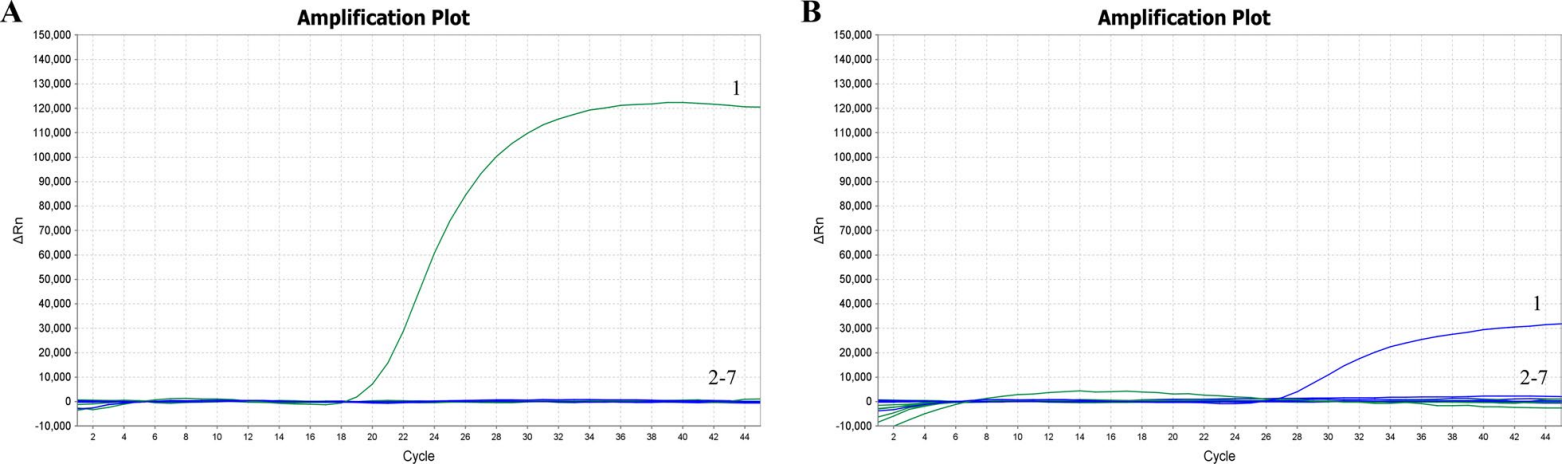
Specificity of the dual probe-specific real-time PCR assay. **A** *T. annulata*-brs (1), other pathogens and negative control (2–7), **B** *T. annulata*-bss (1), and other pathogens and negative control (2–7)

Templates of *T. annulata*-bss, *T. annulata*-brs, *T. sinensis*, *T. orientalis*, *B. bigemina*, and *B. bovis* were used in the dual probe-specific real-time PCR assay. FAM and VIC fluorescent signals were detected only when *T. annulata*-brs-positive clinical samples and *T. annulata*-bss-positive clinical samples were used as templates (Fig. 6). No fluorescent signals were observed for other pathogens that are common in bovine blood, viz. *T. sinensis*, *T. orientalis*, *B. bigemina*, and *B. bovis*. These results confirm the high specificity of the method developed in this study.

### Field sample detection

**Fig. 7:**
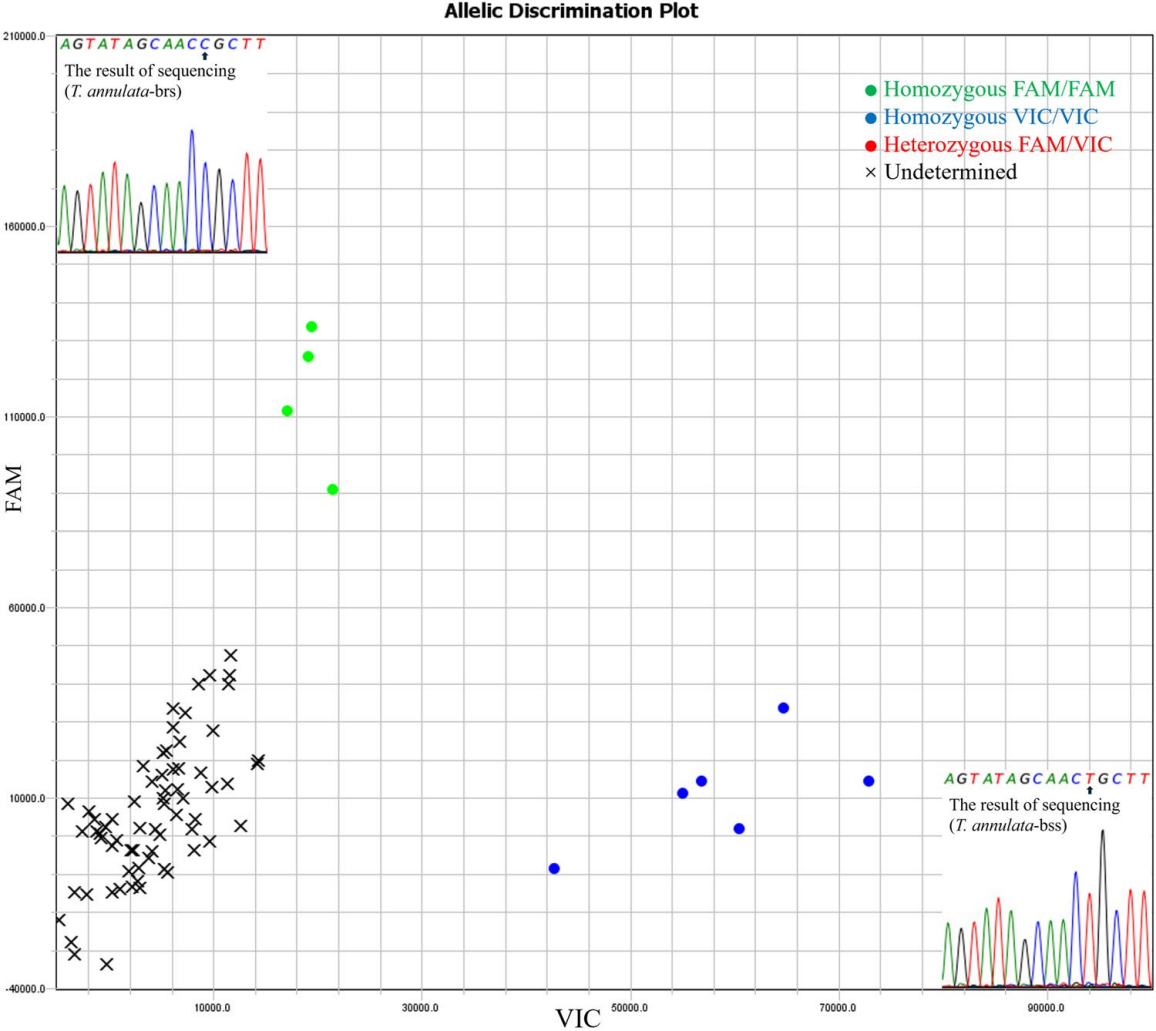
Scatter plot of SNP genotyping results from field sample detection and Sanger sequencing. The green dots (homozygous FAM/FAM) represent *T. annulata*-brs positive, the blue dots (homozygous VIC/VIC) represent *T. annulata*-bss positive, the red dots (heterozygous FAM/VIC) represent coinfection *T. annulata*-bss and *T. annulata*-brs, and the black X (undetermined) represents *T. annulata*-bss and *T. annulata*-brs negative

The results revealed that 92 samples were positive for *T. annulata*-bss and that 23 samples were positive for *T. annulata*-brs (Table 3, Fig. 7.), with no coinfection detected. The overall infection rate of *T. annulata* was 21.7% (115/531), of which *T. annulata*-brs accounted for 20% (23/115) of the total number of infections. A subset of positive samples was randomly selected for validation via Sanger sequencing, and the results were consistent with those obtained via the dual probe-specific assay. Comparison test results revealed an equivalent detection rate for *T. annulata*-brs, whereas the overall detection rate for *T. annulata* was higher than that reported using a previously described method (infection rate = 17.1%, 91/531) [8, 20

**Table 3:** Results of field sample detection

Province	Number of samples	Number of *T. annulata* -bss	Number of *T. annulata* -brs	Number of *T. annulata*-brs Previous method Su et al. [8]	Number of *T. annulata* Previous method Junlong et al. [20]
		Present study			
Xinjiang	227	59	23	23	71
Ningxia	33	4	0	0	0
Qinghai	103	11	0	0	11
Gansu	65	16	0	0	9
Inner Mongolia	53	2	0	0	0
Total	531	92	23	23	91

## Discussion

Although buparvaquone remains a frequently used treatment for bovine tropical theileriosis caused by *T. annulata*, recent genomic surveillance has revealed alarming therapeutic failures in endemic regions. The emergence of point mutations in the *Cytb* and/or *TaPIN1* genes is thought to contribute to the development of buparvaquone resistance [7]. These dual-target mutations likely interfere with buparvaquone binding through steric hindrance, representing a concerning mechanism underlying the evolution of drug resistance.

Although Sanger sequencing remains the gold standard for detecting resistance-associated mutations, its reliance on post-PCR processing is time-consuming and laborious [25]. Recent advances have led to the development of a *TaPIN1*-targeted real-time PCR assay with a detection limit of 2.72 × 10^1^ copies/μl (higher than our method) for identifying *T. annulata* -brs [8]. While this previous method achieved a comparable detection rate in field-collected cattle blood samples (likely because of the high load of *T. annulata* in samples, which is above both assays’ sensitivity thresholds), it has a critical limitation: the inability to distinguish coinfections, which is a common epidemiological challenge in endemic areas. The *Cytb* gene has been shown in previous studies to be a highly specific and sensitive target [26] that is widely used in phylogenetic studies [27], and TaqMan real-time PCR is considered to be one of the most convenient and accurate approaches for SNP genotyping [28]. In this study, we identified six point mutations in the *Cytb* gene in *T. annulata*-brs strains and developed a dual probe-specific real-time PCR assay targeting a 115-bp fragment of the *T. annulata Cytb* gene. The assay employs TaqMan-MGB probes for rapid, one-step differentiation between *T. annulata*-bss (genotype: ACT) and *T. annulata*-brs (genotype: ACC). Probes were labelled at the 5′ end with VIC (*T. annulata*-bss)/FAM (*T. annulata*-brs) dyes and nonfluorescent quenchers (MGB) at the 3′ end to improve sensitivity and specificity. This design enables both genotypes to be detected in a single reaction, making the assay simple to perform and suitable for large-scale genotyping applications.

Molecular surveillance data have revealed that *T. annulata* is the predominant and clinically significant piroplasm species in Chinese cattle and cocirculates with *T. sinensis* and *T. orientalis* along transnational transmission corridors [29]. In this study, we identified a geographically clustered emergence of *T. annulata*-brs in Xinjiang, a strategically linked area of China bordering eight countries, including Pakistan, Kazakhstan, and Tajikistan, through the Tianshan Border Trade Corridor. This epidemiological pattern has emerged despite the absence of buparvaquone use in local livestock, suggesting potential resistance gene introgression through two key mechanisms: transboundary movement of asymptomatic carrier cattle via the Central Asian livestock trade network [30, 31] and vector-mediated spread by *Hyalomma* tick species migrating along seasonal pastures. The 100% concordance between our TaqMan-MGB assay and Sanger sequencing results confirms the assay’s diagnostic robustness. These findings not only validate the assay’s suitability for large-scale resistance monitoring but also raise a critical alert that the expanding presence of drug-resistant *T. annulata* genotypes along the northwestern frontier of China may signal broader antimicrobial resistance proliferation in piroplasmosis-endemic regions across Eurasia.

This multiplex detection platform represents a significant advance over workflows for transboundary resistance surveillance; it enables one-hour single-tube dual-channel detection (48 h for Sanger sequencing) and simultaneous discrimination of wild-type (ACT_ (*T. annulata*-bss)) and mutant (ACC_ (*T. annulata*-brs)) haplotypes at sensitivity thresholds ≤ 10^1^ copies/μl. This operational advantage facilitates real-time geospatial monitoring of resistance alleles, a capability that is particularly critical along China’s 5700-km border with Central Asia.

## Conclusions

We identified six point mutations in the *Cytb* gene and developed a dual probe-specific TaqMan SNP real-time PCR assay for the rapid detection of *Cytb* mutations associated with buparvaquone resistance in *T. annulata*. This assay provides implications for guiding the treatment of tropical theileriosis and for enhancing the epidemiological surveillance of emerging buparvaquone resistance in endemic regions.

## Supplementary Information

Additional file 1

Additional file 2

Additional file 3

## Data Availability

The data supporting the conclusions of this article are included within the article.

## Abbreviations

SNP: Single-nucleotide polymorphism
Cytb: Cytochrome b
MGB: Minor groove binder
TaPIN1: *T. annulata* prolyl isomerase I gene
NCBI: National Center for Biotechnology Information
CAAS: Chinese Academy of Agricultural Sciences
VVBD: Vector and Vector-borne Diseases
