# Supplementary material for: SNP-based molecular diagnostic platform: rapid single-step identification of Theileria annulata and its buparvaquone-resistant strains
Source: Parasit Vectors. 2025 Jul 1;18:247. doi: 10.1186/s13071-025-06884-y (PMC12219782; doi:10.1186/s13071-025-06884-y)
Supplement: Supplementary file 1 — Additional file 1 [file 13071_2025_6884_MOESM1_ESM.docx]

**PCR assay protocol**

All PCR reactions mixtures consisted of 12.5 μL Phanta Max Master Mix (Vazyme, China), 0.8 μM of each primer and 1 μL of DNA template. Finally, nuclease-free water was supplemented to a final volume of 25μL.

Detection of *T. annulata*: amplification was performed on T100 Thermal Cycler (BIO-RAD, USA) and the protocol conditions were: 95 °C for 3 min followed by 35 cycles of denaturation (95 °C for 15 s), primer annealing (56 °C for 15 s) and extension (72 °C for 30 s). The final extension was performed at 72 °C for 5 min.

Amplification of Cytb gene full-length sequences: amplification was performed on T100 Thermal Cycler (BIO-RAD, USA) and the protocol conditions were: 95 °C for 3 min followed by 35 cycles of denaturation (95 °C for 15 s), primer annealing (60 °C for 15 s) and extension (72 °C for 60 s). The final extension was performed at 72 °C for 5 min.
