# Supplementary material for: SNP-based molecular diagnostic platform: rapid single-step identification of Theileria annulata and its buparvaquone-resistant strains
Source: Parasit Vectors. 2025 Jul 1;18:247. doi: 10.1186/s13071-025-06884-y (PMC12219782; doi:10.1186/s13071-025-06884-y)
Supplement: Supplementary file 2 — Additional file 2 [file 13071_2025_6884_MOESM2_ESM.docx]

**Construction of standard plasmid.**

The standard plasmid was constructed using the complete Cytb gene (1092 bp) of *T. annulata*-bss and *T. annulata*-brs. The Cytb genes were amplified and purified by a PCR product purification kit (OMEGA, UK), then cloned into the pGEM-T Easy vector (Progema, USA). The plasmids were transformed into *E. coli* DH5α competent cells (TaKaRa Biotech Co., Ltd., Dalian). The plasmids were extracted using a Plasmid Mini Kit (OMEGA, UK) and sequenced by outsourcing from Beijing Tsingke Biotech Co., Ltd. (Beijing, China). The standard plasmids were labeled as T-XJS / T-NM1 respectively and stored at -80℃.
