# Supplementary material for: SNP-based molecular diagnostic platform: rapid single-step identification of Theileria annulata and its buparvaquone-resistant strains
Source: Parasit Vectors. 2025 Jul 1;18:247. doi: 10.1186/s13071-025-06884-y (PMC12219782; doi:10.1186/s13071-025-06884-y)
Supplement: Supplementary file 3 — Additional file 3 [file 13071_2025_6884_MOESM3_ESM.pdf]

Editing Certificate

This document certifies that the manuscript

A SNP-based molecular diagnostic platform: rapid single-step identification of Theileria annulata and its Buparvaquone-resistant strains

prepared by the authors

Jin Che, Yijun Chai, Shuaiyang Zhao, Jinming Wang, Jianxun Luo, Guiquan Guan, Hong Yin, Wei Li

was edited for proper English language, grammar, punctuation, spelling, and overall style by one or more of the highly qualified English speaking editors at AJE.

This certificate was issued on **May 30, 2025** and may be verified on the AJE website using the verification code **9647-C18C-AC88-FB49-2EB0** .

Neither the research content nor the authors' intentions were altered in any way during the editing process. Documents receiving this certification should be English-ready for publication; however, the author has the ability to accept or reject our suggestions and changes. To verify the final AJE edited version, please visit our verification page at aje.com/certificate. If you have any questions or concerns about this edited document, please contact AJE at support@aje.com.

AJE provides a range of editing, translation, and manuscript services for researchers and publishers around the world. For more information about our company, services, and partner discounts, please visit aje.com.
